# Supplementary material for: Identification and functional analysis of non-coding regulatory small RNA FenSr3 in Bacillus amyloliquefaciens LPB-18
Source: PeerJ. 2023 May 15;11:e15236. doi: 10.7717/peerj.15236 (PMC10194069; doi:10.7717/peerj.15236)
Supplement: Supplemental Information 4 [file peerj-11-15236-s004.zip › KO/CK-vs-T1_map/map04927.html]

KEGG PATHWAY: Cortisol synthesis and secretion - Reference pathway


|  |  |
| --- | --- |
| **Cortisol synthesis and secretion - Reference pathway** |  |

[
Pathway menu
| Organism menu
| Pathway entry
| Show description
| User data mapping
]

Cortisol is the main endogenous glucocorticoid, which affects a plethora of physiological functions, e.g., lipid and glucose metabolism, metabolic homeostasis and adaptation to stress. Cortisol production is primarily regulated by corticotropin (ACTH) in zona fasciculata. The stimulatory effect of ACTH on cortisol synthesis depends on cAMP dependent signaling, but also involves membrane depolarization and increased cytosolic Ca2+. Each of cAMP and Ca2+ induces the expression of StAR, stimulating intramitochondrial cholesterol transfer, as well as the steroidogenic enzymes in the pathway from cholesterol to cortisol (e.g., CHE, CYP17A1, CYP11B1).

|  |  |  |
| --- | --- | --- |
| Reference pathway | 184% 150% 122% 100% 82% 67% 55% | 图片下载 |
